# Regulatory Effect of *Lactiplantibacillus plantarum* 2-33 on Intestinal Microbiota of Mice With Antibiotic-Associated Diarrhea

**DOI:** 10.3389/fnut.2022.921875

**Published:** 2022-06-09

**Authors:** Wuyundalai Bao, Yuxing He, Jinghe Yu, Mingchao Liu, Xiaofeng Yang, Na Ta, Enxin Zhang, Chengyuan Liang

**Affiliations:** College of Food Science and Engineering, Inner Mongolia Agricultural University, Hohhot, China

**Keywords:** *Lactiplantibacillus plantarum*, antibiotic-associated diarrhea, mice, intestinal flora, regulating function, inflammatory cytokines

## Abstract

Diarrhea is one of the common adverse reactions in antibiotic treatment, which is usually caused by the imbalance of intestinal flora, and probiotics play an important role in the structure of intestinal flora. Therefore, this experiment studied the regulatory effect of *Lactiplantibacillus plantarum* 2-33 on antibiotic-associated diarrhea (AAD) mice. First, the AAD mice model was established by the mixed antibiotic solution of gentamicin sulfate and cefradine. Then, the physiological indexes and diarrhea of mice were observed and recorded by gastric perfusion of low dose (1.0 × 10^7^ CFU/ml), medium dose (1.0 × 10^8^CFU/ml), and high dose (1.0 × 10^9^ CFU/ml) strain 2-33. 16S rRNA gene V3-V4 regions were sequenced in colon contents of mice in control group, model group, self-healing group, and experimental group, respectively, and the diversity of intestinal flora and gene function prediction were analyzed. The results showed that the intestinal flora of AAD mice was not significantly regulated by gastric perfusion of strain 2-33 to 7 days, but the relative abundance and diversity of intestinal flora of AAD mice were significantly improved by gastric perfusion to 14 days (*p* < 0.05). In addition, at the genus level, the relative abundance of *Lactobacillus* increased significantly, and the relative abundance of *Enterococcus* and *Bacillus* decreased significantly (*p* < 0.05). In addition, the regulation of strain 2-33 on intestinal flora of AAD mice was time- and dose-dependent, short-term gastric perfusion, and low dose had no significant effect (*p* > 0.05). Strain 2-33 can significantly increase the levels of anti-inflammatory cytokines IL-4 and IL-10, significantly decrease the levels of proinflammatory cytokines TNF-α and IFN-γ (*p* < 0.05), and can also adjust carbohydrate metabolism, amino acid metabolism, and energy metabolism to normal levels, thus accelerating the recovery of intestinal flora structure of AAD mice. In summary, strain 2-33 can improve the structure and diversity of intestinal flora of AAD mice, balance the level of substance and energy metabolism, and play a positive role in relieving diarrhea, maintaining and improving the intestinal microecological balance.

## Introduction

Antibiotics are the main drugs for the treatment of various bacterial infections and diseases, but long-term and large-scale use of antibiotics will increase the risk of clinical complications, such as antibiotic-associated diarrhea (AAD) (1), which is one of the common adverse reactions of antibiotic therapy. It is characterized by intestinal flora disorder, decrease in intestinal short-chain fatty acid (SCFA) concentration, accumulation of carbohydrates in lumen and bile acid in colon, and change of water absorption (2, 3), which eventually leads to diarrhea. Clinical mild AAD is mainly manifested by the change in stool frequency and shape, whereas severe AAD will lead to a large number of special conditioned pathogens (4). However, the etiology of AAD is still unclear. At present, the destruction of intestinal flora is considered as the key factor, especially the infection of *Clostridium difficile* can lead to a series of diseases, from occasional diarrhea to colitis, toxic megacolon, and potential death (5). Among patients receiving antibiotic treatment, the incidence of AAD is about 5–35%, and it varies with different groups and types of antibiotic users. The occurrence time ranges from a few hours after the antibiotic treatment starts to 6–8 weeks after it stops (6).

Intestinal flora plays a vital role in human health and is named “forgotten organ.” Intestinal flora and its metabolites are considered as an important part of human physiology (7). The number of genes in the intestinal flora is about 100 times that of the human genome, and the human gastrointestinal tract can hold nearly 10^14^ microorganisms, almost three times the total number of human cells. From the physiological point of view, microbiome accounts for about 2% of adult body weight, which is equivalent to the size of human brain or liver (8). It is a complex ecosystem, in which there is great interdependence and interference between microbial species and their hosts (9). Therefore, human body health is closely related to the changes of intestinal flora (10). The occurrence of AAD will affect the ability of resident microorganisms to resist the invasion of pathogenic microorganisms (11), or the excessive growth of pathogenic bacteria existing in the flora, thus affecting the relative abundance and diversity of intestinal flora (12). The imbalance of intestinal flora can affect the immune function and structural integrity of intestinal mucosa, leading to the weakening of intestinal mucosal barrier function, and the colonization and reproduction of foreign pathogenic bacteria in the intestine to become the dominant flora, eventually leading to diarrhea or enteritis. Although modern treatments have certain effects, they cannot repair the damaged mucosal barrier or regulate the intestinal flora (13). Even after the total number of bacteria is restored, it will have a lasting impact on the balance of intestinal flora, thus affecting patients’ susceptibility to other diseases (14). According to the reported research, the combination of *Lactobacillus reuteri* LRE02 and *Lactobacillus rhamnosus* LR04 can effectively improve infant diarrhea caused by antibiotics (15).

*Lactobacillus* strains are considered as one of the most important probiotics (16), and they are the prominent bacteria in human intestinal flora and have become the most commonly used probiotics in human medicine (including obstetrics) (17). Among them, *Lactiplantibacillus plantarum* exists in human gastrointestinal tract, and it can produce a variety of natural antibacterial compounds, such as organic acids, bacteriocins, hydrogen peroxide, and diacetyl, to maintain the balance of intestinal flora, balance the immune response *in vivo*, and promote the absorption of nutrients (18). Therefore, this experiment took *Lactiplantibacillus plantarum* 2-33, which has a probiotic effect, as the research object, to study the effects of its gastric perfusion days and dosage on the physiological characteristics, inflammatory cytokines, and intestinal flora of AAD mice. The research results not only enrich the scientific understanding of the probiotic effect of strain 2-33 but also provide the basis for the application of probiotic effect of strain 2-33.

## Materials and Methods

### Experimental Reagents and Strains

Gentamicin sulfate injection (2.0 ml) and cefradine capsule (0.25 g) were obtained from Shandong Lu Kang Pharmaceutical Group Saite Co., Ltd. (batch no: 20081001) and Zhuhai Federal Pharmaceutical Co., Ltd., Zhongshan Branch, respectively (batch no: 01063205). ELISA kit was purchased from Jiangsu Meibiao Biotechnology Co., Ltd. The mixed antibiotic solution with a concentration of 62.5 g/L was prepared with gentamicin sulfate and cefradine, preparation when used.

*Lactiplantibacillus plantarum* 2-33 is derived from Mongolia koumiss samples, and the previous research shows that it has excellent probiotic effects (19). The viable count of strain 2-33 bacterial powder (1.0 × 10^9^ CFU/g) was adjusted to 1.0 × 10^7^ CFU/mL (low dose), 1.0 × 10^8^ CFU/ml (medium dose), and 1.0 × 10^9^ CFU/ml (high dose) bacterial suspension with sterile saline before the experiment.

### Animal Ethics Statement

A total of 60 male SPF KM mice (6–8 weeks old, 41.30 ± 0.76 g) were purchased from SPF (Beijing) Biotechnology Co., Ltd. [license no: SCXK (Beijing) 2019-0010, Beijing, China] and kept at a temperature of 24.0 ± 1.0 °C and a humidity of 50 ± 5.0%, 12-h light/dark cycle and filtered air. Then, 5 days before the start of the experiment is the adaptation period, and mice can get free access to food and water. All animal experiments strictly follow the guidelines for the care and use of laboratory animals described by the National Institutes of Health, and every effort is made to maximize the health of mice, minimize their pain, and minimize the number of animals used.

### Experimental Grouping and Establishment of Antibiotic-Associated Diarrhea Mice Model

After 5 days of adaptation, the mice were divided into control group, model group, self-healing group, and experimental group according to the principle of equal average weight among groups, and the experimental group included low-dose group, middle-dose group, and high-dose group, with a total of 60 mice. In the experimental period, except the control group, the rest groups were given mixed antibiotic solution (62.5 g/L) of 10 ml/kg/time, two times a day (AM9:00 and PM5:00) for 5 days. The control group was given the same volume of normal saline. The mice in model group, self-healing group, and experimental group showed stool thinning and softening, defecation times increased, dirty anus, dull back hair, and slow movement, which confirmed that the model was successful and reached the model standard (20). After the successful establishment of the AAD model, the mice in the model group (A) were free drinking, but after fasting for 12 h, they were anesthetized with 3% pentobarbital sodium (45 mg/kg) and then dissected for blood collection, and the colon contents were collected and stored at –80 °C (21). The rest of the groups entered the experimental period.

### Gastric Perfusion Dosage of Bacterial Suspension of Strain 2-33 and Collection Scheme of Colon Contents

Table 1 shows the code of each group, the scheme of gastric perfusion, and the collection time of test samples during the experiment. The gastric perfusion dose of each mouse is calculated as 10 ml/kg/day, and the specific scheme is as follows: the control group and the self-healing group were given gastric perfusion saline during the experiment, and the experimental group was given gastric perfusion suspension of strain 2-33 with low dose, medium dose, and high dose, respectively. Samples of colon contents of mice in each group were collected on the 7th day and 14th day of the experiment. During the whole experiment, feed composition, feeding environment, and feeding mode remained unchanged.

**TABLE 1:** The mice’s plan of gastric perfusion of strain 2-33 and the sampling time of samples.

Group (code)	Gastric perfusion scheme	Test sample collection time		
		Established the AAD model	7th day	14th day
Control group(C)	Normal saline	C		
Model group(A)	Mixed antibiotic solution	A		
Self-healing group(S)	Normal saline		S7	S14
Low dose group(L)	1.0 × 10^7^ CFU/mL (Strain 2-33 bacterial suspension)		L7	L14
Medium dose group(M)	1.0 × 10^8^ CFU/mL (Strain 2-33 bacterial suspension)		M7	M14
High dose of groups(H)	1.0 × 10^9^ CFU/mL (Strain 2-33 bacterial suspension)		H7	H14

### Detection and Treatment of Physiological Characteristics of Antibiotic-Associated Diarrhea Mice

During the experiment, every other day at the same time (AM8:00), according to Table 2 (22), the mice in each group were scored for diarrhea, the physiological characteristics (exercise state, stool hardness, and defecation times) were observed, and their weight (g), food intake (g), and water consumption (ml) were measured. The test sample collection time is shown in Table 1. The mice were fasted 12 h before the sample collection, but the water was not stopped. A total of three mice in each group were randomly anesthetized with 3% pentobarbital sodium (45 mg/kg), and then dissected for blood collection, and samples of colon contents were collected and stored at −80 °C (21).

**TABLE 2:** AAD mice diarrhea score standard.

Score	Diarrhea status
0	Normal
1	Soft stool, decreased spontaneous activity, and weight gaining slows down
2	Wet stool, perianal contamination, decreased spontaneous activity, and weight loss

### Determination of Inflammatory Cytokines in Serum of Antibiotic-Associated Diarrhea Mice

According to the manufacturer’s instructions, the contents of interleukin-4 (IL-4), interleukin-10 (IL-10), tumor necrosis factor-α (TNF-α), and interferon-γ (IFN-γ) in mice serum were determined by ELISA kit.

### Analysis of Bacterial Diversity of Intestinal Flora

#### DNA Extraction and the PCR Amplification of 16S rRNA Genes

Microbial community genomic DNA was extracted from mice colonic content samples using the E.Z.N.A.^®^ soil DNA Kit (Omega Bio-Tek, Norcross, GA, United States) according to the manufacturer’s instructions. The DNA extract was checked on 1% agarose gel, and DNA concentration and purity were determined with NanoDrop 2000 UV-vis spectrophotometer (Thermo Scientific, Wilmington, NC, United States). The hypervariable regions V3–V4 of the bacterial 16S rRNA genes were amplified with primer pairs 338F (5′-ACTCCTACGGGAGGCAGCAG-3′) and 806R (5′-GGACTACHVGGGTW TCTAAT-3′) by an ABI GeneAmp^®^ 9700 PCR thermocycler (ABI, CA, United States). The PCR amplification of 16S rRNA genes was performed as follows: initial denaturation at 95°C for 3 min, followed by 27 cycles of denaturing at 95°C for 30 s, annealing at 55°C for 30 s, extension at 72°C for 45 s, single extension at 72°C for 10 min, and end at 4°C. The PCR mixtures contain 5 × *TransStart* FastPfu buffer 4 μl, 2.5 mM dNTPs 2 μL, forward primer (5 μM) 0.8 μl, reverse primer (5 μM) 0.8 μl, *TransStart* FastPfu DNA Polymerase 0.4 μl, template DNA 10 ng, and finally ddH_2_O up to 20 μl. PCRs were performed in triplicate. The PCR product was extracted from 2% agarose gel and purified using the AxyPrep DNA Gel Extraction Kit (Axygen Biosciences, Union City, CA, United States) according to the manufacturer’s instructions and quantified using Quantus™ Fluorometer (Promega, United States).

#### Illumina MiSeq Sequencing

Purified amplicons were pooled in equimolar and paired-end sequenced on an Illumina MiSeq PE300 platform/NovaSeq PE250 platform (Illumina, San Diego, CA, United States) according to the standard protocols by Majorbio Bio-Pharm Technology Co. Ltd. (Shanghai, China). The raw reads were deposited into the NCBI Sequence Read Archive (SRA) database (accession number: PRJNA827246).

#### Processing of Sequencing Data

The raw 16S rRNA gene sequencing reads were demultiplexed, quality-filtered by FASTp version 0.20.0 (23), and merged by FLASH version 1.2.7 (24) with the following criteria: (i) the 300 bp reads were truncated at any site receiving an average quality score of < 20 over a 50-bp sliding window, the truncated reads shorter than 50 bp were discarded, and reads containing ambiguous characters were also discarded; (ii) only overlapping sequences longer than 10 bp were assembled according to their overlapped sequence. The maximum mismatch ratio of overlap region is 0.2. Reads that could not be assembled were discarded; (iii) samples were distinguished according to the barcode and primers, and the sequence direction was adjusted, exact barcode matching, 2 nucleotide mismatch in primer matching.

Operational taxonomic units (OTUs) with 97% similarity cutoff were clustered using UPARSE version 7.1 (25, 26), and chimeric sequences were identified and removed. The taxonomy of each OTU representative sequence was analyzed by RDP Classifier version 2.2 (27) against the 16S rRNA database (e.g., Silva v138) using a confidence threshold of 0.7.

### Data Analysis

Mothur software (v.1.30.2) was used to calculate the alpha diversity index of OTU level, such as Ace, Chao, and coverage index, and draw the dilution curve. Beta diversity distance matrix was calculated by Qiime software, and PCoA and NMDS diagrams were drawn, respectively. Through LEfSe method analysis, the species with significant differences in abundance among different groups were found, and the final difference species were obtained by linear discriminant analysis (LDA). The gene function was predicted and analyzed by PICRUSt2 software, and the relative abundance heat map of functional modules was drawn to find out KEGG metabolic pathway involved by intestinal microbes. All the above drawings are made with R language tools.

### Statistical Analysis

The experimental data were expressed as $\bar{\text{x}}$ ± SD. SPSS 26 software was used for statistical analysis, and Origin 2020b was used for drawing. After one-way analysis of variance (ANOVA), statistical analysis between groups was performed by Duncan’s multiple range tests, and the differences were considered as significance at *p* < 0.05. Bioinformatic analysis is carried out on Majorbio I-Sanger cloud platform.

## Results

### The Gastric Perfusion Dosage of Bacterial Suspension of Strain 2-33 and the Collection Scheme of Colon Contents

**FIGURE 1:**
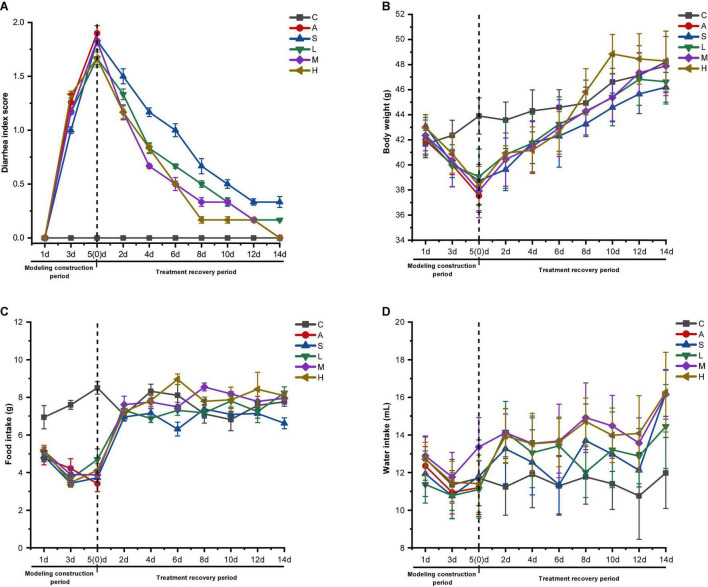
Effect of strain 2-33 on physiological characteristics of AAD mice. Diarrhea index score **(A)**; body weight **(B)**; food intake **(C)**; water intake **(D)**. C, control group; A, model group; S, self-healing group; L, low-dose group; M, middle-dose group; H, high-dose group. The experimental results are expressed as the $\bar{\text{x}}$ ± SD, *n* = 6.

The AAD mice model was established by gavage of mixed antibiotic solution (62.5 g/L) at the standard of 10 ml/kg/time for 5 days, two times a day. As can be seen from Figure 1, on the 5th day, the diarrhea scores and physiological characteristics of mice in model group, self-healing group, and experimental group showed obvious changes compared with the control group. In particular, the diarrhea score reached the highest value, and the mice showed sluggish movement, soft or watery stool, increased defecation frequency, sharp weight loss, decreased food intake, and increased water consumption, so the AAD mice model was successfully established.

From the diarrhea score of mice (Figure 1A), it can be seen that the control group mice did not produce diarrhea during the experimental period, so the diarrhea score value is 0; however, the diarrhea score of the experimental group decreased rapidly after the gastric perfusion of strain 2-33, and the diarrhea score of the self-healing group also decreased rapidly, but the decline rate was obviously lower than that of the experimental group. On the 14th day, the diarrhea scores of the middle and high dose groups reached the normal values of the control group. There is a certain difference in diarrhea score between self-healing group and low-dose group and the control group, but the difference between self-healing group and low-dose group is larger. Therefore, it is determined that strain 2-33 has a positive effect in curing diarrhea in AAD mice, and the gastric perfusion dosage has a significant effect on the speed of curing diarrhea.

From the change of body weight (Figure 1B) and food intake (Figure 1C) of mice, it can be seen that gastric perfusion of strain 2-33 increased the food intake and body weight of AAD mice day by day. On the 14th day, the weight of the middle and high dose groups recovered to the control group level. Compared with the control group, the weight of self-healing group and low-dose group has a certain difference, but the difference in self-healing group is bigger. This fully proves that strain 2-33 has a positive effect on the physical recovery of AAD mice, which is consistent with the diarrhea score (Figure 1A).

From the change of water intake (Figure 1D), it can be seen intuitively that compared with the control group, the water intake of self-healing group and experimental group increased, and the water intake of experimental group mice was obviously larger than that of self-healing group, which proved that strain 2-33 accelerated the cure rate of diarrhea in AAD mice, and the cure rate was faster than that of self-healing group, which was related to the decrease of diarrhea score, the increase of food intake, and weight of AAD mice. Therefore, the recovery of physiological indexes of AAD mice by gastric perfusion of strain 2-33 is better than that of self-healing group, and strain 2-33 can cure diarrhea of AAD mice.

### Effect of Strain 2-33 on Serum Inflammatory Cytokines

**FIGURE 2:**
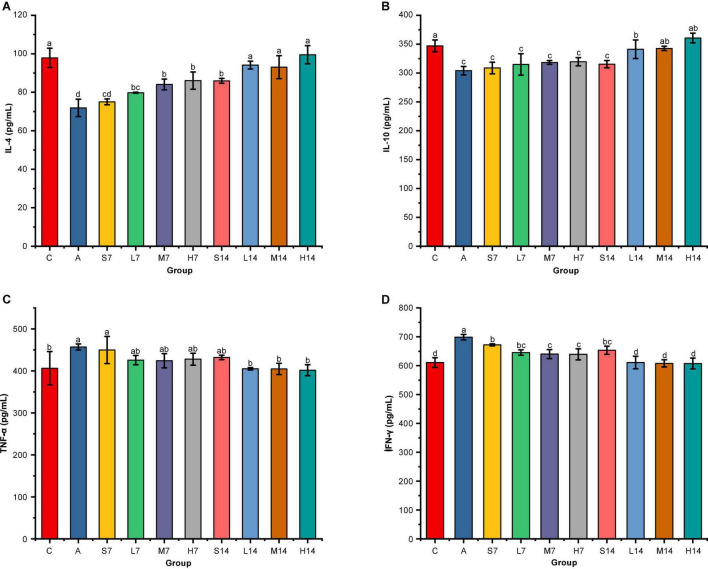
Effect of strain 2-33 on cytokines in serum of AAD mice. IL-4 cytokines **(A)**; IL-10 cytokines **(B)**; TNF-α cytokines **(C)**; IFN-γ cytokines **(D)**. There are significant differences among different letters (*p* < 0.05).

According to the effect of strain 2-33 on inflammatory cytokines in serum of AAD mice (Figure 2), compared with the control group, the levels of anti-inflammatory cytokines (IL-4 and IL-10) in the model group decreased significantly (*p* < 0.05), whereas the levels of proinflammatory cytokines (TNF-α and IFN-γ) increased significantly (*p* < 0.05). It was proved that gastric perfusion of mixed antibiotics solution caused intestinal inflammation in mice, resulting in the changes in the levels of inflammatory cytokines in AAD mice. On the 7th day, compared with the self-healing group (S7), the levels of anti-inflammatory cytokines in the experimental group all increased to some extent, except that the IL-4 of the middle-dose group (M7) and the high-dose group (H7) was significantly higher than those in the self-healing group (S7) (*p* < 0.05), and there was no significant difference in other groups (*p* > 0.05). On the 14th day, compared with the self-healing group (S14), the levels of anti-inflammatory cytokines in the experimental group were significantly increased (*p* < 0.05), and the levels of anti-inflammatory cytokines (IL-4 and IL-10) in other experimental groups were not significantly different from those in the control group (*p* > 0.05). On the 7th day, compared with the self-healing group (S7), the levels of proinflammatory cytokines in the experimental group decreased to some extent, especially in the middle-dose group (M7) and high-dose group (H7) of IFN-γ, compared with the self-healing group (S7) (*p* < 0.05). On the 14th day, compared with the self-healing group (S14), the level of TNF-α factor had no significant difference (*p* > 0.05), but the level of IFN-γ decreased significantly (*p* < 0.05). Moreover, on the 14th day, the levels of proinflammatory cytokines (TNF-α and IFN-γ) were adjusted to those of the control group, and there was no significant difference between them (*p* > 0.05), but the levels of pro-inflammatory cytokines in the high-dose group were closer to those of the control group.

Therefore, it is proved that strain 2-33 has a significant effect on the level of inflammatory cytokines in serum of AAD mice, and the days of gastric perfusion have the most significant effect. Although the influence of different days of gastric perfusion of TNF-α is not significant (*p* > 0.05), the influence of other inflammatory cytokines is significant (*p* < 0.05), but the influence of gastric perfusion on TNF-α gradually increases with the prolongation of gastric perfusion. Moreover, with the extension of the days of gastric perfusion, the greater the dose, the greater the impact and the more significant the difference.

### Evaluation of Sequencing Data Quality and Analysis of Bacterial Alpha Diversity Among Groups

**FIGURE 3:**
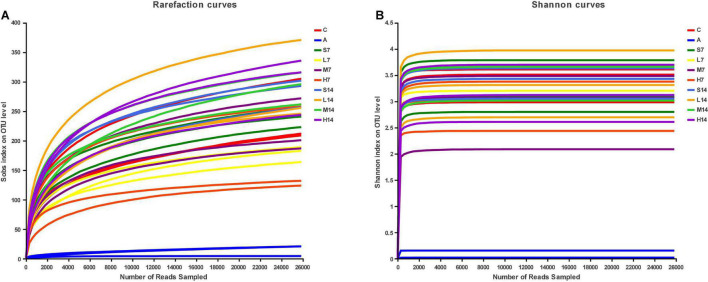
Rarefaction curves **(A)** and Shannon index curves **(B)**.

By drawing the rarefaction curves and Shannon index curves, the sequencing quality and depth of 16S rRNA genes V3–V4 region in the colon content of AAD mice were evaluated. From the curve of Sob index (Figure 3A), it can be seen that the number of bacterial OTU in mice colon content increases with the increase of sequencing depth and finally tends to be flat; Shannon index curve (Figure 3B) reached a stable plateau, and the coverage values were all higher than 0.99 (Table 3), which indicated that the sequencing quantity of 16S rRNA genes in colon contents of AAD mice in this study met the requirements of subsequent bioinformatic analysis.

**TABLE 3:** Analysis of alpha diversity of intestinal flora bacteria.

Group	Alpha diversity		
	Ace	Chao	Coverage
C	322.85 ± 49.76^a^	308.98 ± 64.79^ab^	0.9977 ± 0.0361^d^
A	45.69 ± 34.55^d^	31.11 ± 21.32^d^	0.9997 ± 0.0208^a^
S7	299.56 ± 57.28^ab^	289.68 ± 54.06^abc^	0.9982 ± 0.0451^cd^
L7	210.16 ± 10.77^bc^	222.39 ± 6.09^bc^	0.9986 ± 0.0006^bc^
M7	250.61 ± 58.02^abc^	251.97 ± 56.30^abc^	0.9985 ± 0.0493^bcd^
H7	190.00 ± 82.90^c^	189.74 ± 84.21^c^	0.9990 ± 0.0416^ab^
S14	331.04 ± 18.48^a^	343.39 ± 11.48^a^	0.9979 ± 0.0252^cd^
L14	336.81 ± 76.66^a^	351.43 ± 73.01^a^	0.9978 ± 0.0586^cd^
M14	340.29 ± 35.32^a^	337.06 ± 42.80^a^	0.9978 ± 0.0458^cd^
H14	345.04 ± 62.29^a^	351.76 ± 71.44^a^	0.9978 ± 0.0586^cd^

*$\bar{\text{x}}$ ± SD; within each column; values followed by different letters are significantly different from each other (p < 0.05).*

According to the Ace index and Chao index (Table 3), compared with the control group, the Ace index and Chao index of the model group decreased significantly (*p* < 0.05), which indicated that the diversity of bacterial alpha in intestinal flora of AAD mice decreased significantly, and further suggested that the AAD mice model was successfully established. On the 7th day, compared with the model group, the Ace index and Chao index of the self-healing group (S7) and the experimental group increased significantly (*p* < 0.05), which indicated that the bacterial alpha diversity of AAD mice intestinal flora increased rapidly. On the 14th day, there was no significant difference in Ace index and Chao index between the self-healing group and the experimental group and the control group (*p* > 0.05), which proved that the bacterial alpha diversity of AAD mice intestinal flora was fully restored. Moreover, we found that the influence of normal saline on the alpha diversity of AAD mice intestinal flora was greater than that of strain 2-33 when the days of gastric perfusion were short. This phenomenon has never been reported, so it is necessary to verify it in later studies. However, with the extension of the days of gastric perfusion, the positive influence of strain 2-33 gradually became prominent. Therefore, it is determined that the gastric perfusion dose and gastric perfusion days of strain 2-33 have influence on the bacterial alpha diversity.

### Comparative Analysis of Bacterial Beta Diversity Among Samples

**FIGURE 4:**
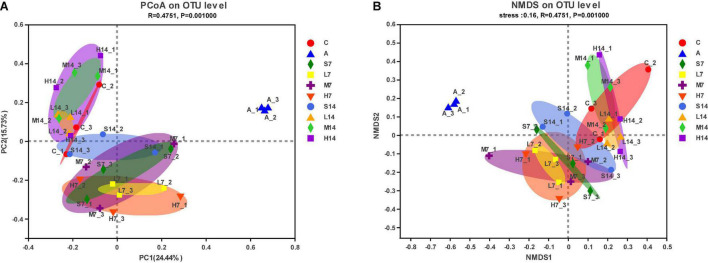
Analysis of bacterial beta diversity of intestinal flora. PCoA figure **(A)**; NMDS figure **(B)**.

To understand the overall difference in intestinal flora diversity among different groups, based on Brary–Curtis distance algorithm, PCoA figure and NMDS figure are drawn. The more similar the sample composition is, the closer the distribution distance is in the diagram. From the PCoA figure (Figure 4A), it can be seen that the successful establishment of AAD mice model leads to the significant separation and distance between the model group and other groups, which indicates that the mixed antibiotic solution by gastric perfusion has caused significant changes in intestinal flora of mice. On the 7th day, the self-healing group (S7) and the experimental group tended to be in the same area in the figure, which indicates that there is a certain similarity in flora composition, but it is more discrete than the control group. On the 14th day, the cross-aggregation degree of the experimental group and the control group increased further, and they gathered in the same area with a smaller area. However, the aggregation of the self-healing group (S14) and the control group was lower than that of the experimental group, and the dispersion was obvious, which indicated that strain 2-33 was helpful to the recovery of intestinal flora of AAD mice. The distribution of NMDS map (Figure 4B) is highly similar to that of PCoA figure, which further proves that strain 2-33 has a good regulatory effect on the bacterial diversity of intestinal flora of AAD mice, and the number of days and dosage of gastric perfusion have significant effects on the regulation of intestinal flora structure of AAD mice.

### Analysis of the Difference Between Groups of Intestinal Flora Composition

**FIGURE 5:**
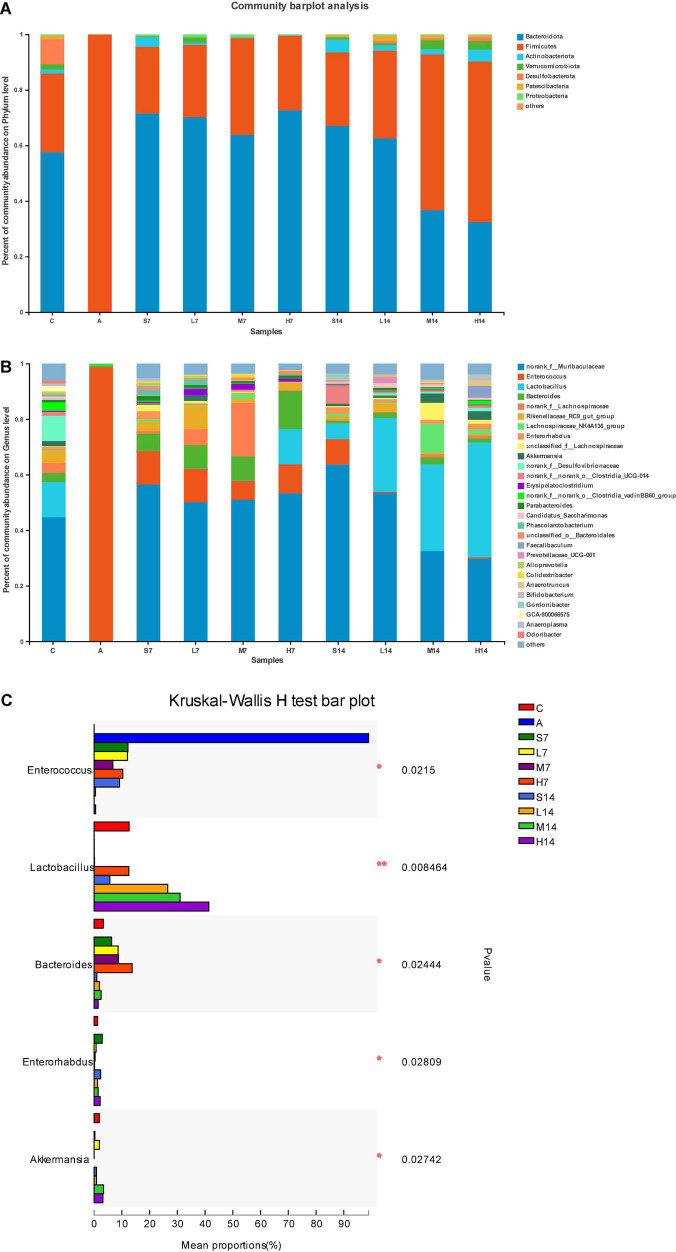
Strain 2-33 regulates the changes of AAD mice flora. The relative abundance of bacterial communities is at phylum level **(A)** and genus level **(B)**; significant difference of the relative abundance of genus level in the top 5 **(C)**. *(*p* < 0.05); **(*p* < 0.01).

The dominant species and their proportion in each group can be known by drawing the community bar diagram. As shown in Figure 5A, the intestinal flora of mice in the control group are mainly Bacteroidota, Firmicutes, Actinobacteriota, Verrucomicrobia, Desulfobacteriota, Patescibacteria, and Proteobacteria at the phylum level. Among them, Bacteroidota and Firmicutes are the dominant phylum, the sum of which is over 90%. Due to the influence of the mixed antibiotic solution in the model group mice, the structure of intestinal flora became single Firmicutes; on the 7th day, the flora diversity of self-healing group (S7) and experimental group increased, and there was a big difference between them and the control group. However, on the 14th day, compared with the self-healing group (S14), the relative abundance of Bacteroidota in the experimental group decreased, the relative abundance of Firmicutes increased, the ratio of Firmicutes and Bacteroidota significantly increased, and the relative abundance of phylum also increased.

As can be seen from Figures 5B,C, at the genus level, the bacterial diversity of AAD mice’s intestinal flora structure has been seriously changed in the model group, with only *Enterococcus* accounting for about 99%; on the 7th day, the *Enterococcus* of the self-healing group (S7) and the experimental group decreased significantly (*p* < 0.05), and other bacteria also recovered significantly, among which *Bacteroides* had high relative abundance in each group, and the high-dose group (H7) contained *Lactobacillus*, whereas the self-healing group (S7), low-dose group (L7), and middle-dose group (L7) were no *Lactobacillus*. On the 14th day, compared with the self-healing group (S14), the *Enterococcus* in the experimental group decreased significantly (*p* < 0.05), whereas *Lactobacillus* became the dominant genus, its relative abundance was better than that in the control group, and it was proportional to the gastric perfusion dose of strain 2-33. With the prolongation of the days of gastric perfusion, the bacterial diversity of intestinal flora in mice was further restored, and the *Bacteroides* with high relative abundance on the 7th day also returned to the level of the control group. Norank-f-*Desulfovibrionaceae* appeared in the control group, which was not found in the self-healing group or the experimental group. The bacterium may have been completely killed by the mixed antibiotic solution without recovery, or the relative abundance of recovery was low and attributed to others.

Therefore, strain 2-33 has a positive effect on the regulation of intestinal flora diversity of AAD mice, and it reflects that the intestinal flora of AAD mice is in a dynamic change of interaction, and the number of days and dosage of gastric perfusion have the effects on the regulation of intestinal flora diversity of AAD mice.

### Analysis of Intestinal Flora Difference

**FIGURE 6:**
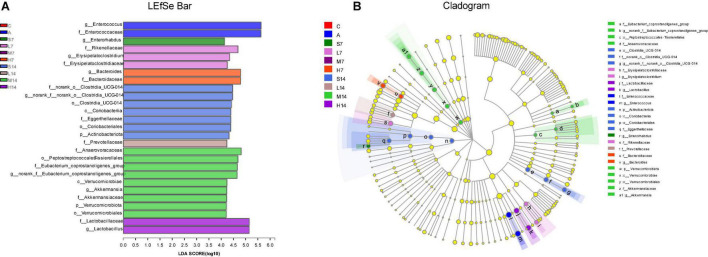
Different analyses of intestinal flora in mice. **(A)** Bar chart of LDA discrimination result, in which LDA value is > 2.0, and the length of bar chart represents LDA value. **(B)** LEfSe hierarchical tree diagram of multi-level species, showing the classification of intestinal flora and its main bacterial structure. In the diagram, the circles radiating from inside to outside are phylum, class, order, family, genus, etc. At different classification levels, each small circle represents a classification at that level. The diameter of the small circle is proportional to the relative abundance. Species with significant differences are colored according to groups, whereas those without significant differences are yellow.

The species with statistically significant differences in relative abundance of bacterial diversity among groups were analyzed by LEfSe, and the biggest difference in flora classification among groups was displayed. The results are shown in Figures 6A,B, which are at genus level, *Enterococcus* was main differential microbiota in the A group; *Enterorhabdus* was main differential microbiota in the S7 group; *Erysipelatoclostridium* was main differential microbiota in the L7 group; *Bacteroides* was main differential microbiota in the H7 group; norank-f-norank-o*-Clostridia-*UCG*-*014 was main differential microbiota in the S14 group; *Akkermansia* and norank-f-*Eubacterium*- *coprostanoligenes* group were main differential microbiota in the M14 group; *Lactobacillus* was main differential microbiota in the H14 group. Therefore, the number of days and dosage of gastric perfusion of strain 2-33 can cause the dynamic changes of significantly different species in intestinal flora of AAD mice.

### Prediction and Analysis of Genes Function of Intestinal Flora

**FIGURE 7:**
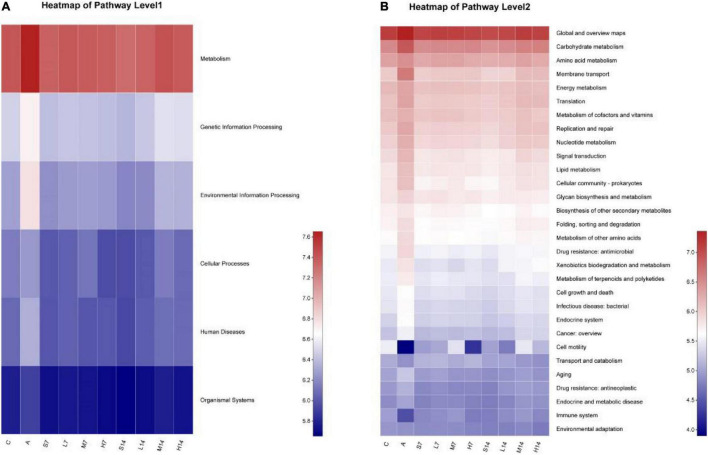
Prediction and analysis of genes function relative abundance based on PICRUSt2 analysis. **(A)** KEGG pathway level 1; **(B)** KEGG pathway level 2.

According to the diversity analysis of intestinal flora of AAD mice, it is known that mixed antibiotic solution will destroy the microecological balance of intestinal flora of mice. Therefore, combined with the data of intestinal flora of mice, the KEGG metabolic pathway involved in it was found out. It can be seen from Figure 7A that at level 1, the genes’ functional abundance of metabolism, genetic information processing, environmental information processing, cellular processes, human diseases, and organic systems in the model group is higher than that in other groups; on the 7th day, compared with the model group, the relative abundance of genes function in the experimental group began to decrease. On the 14th day, the genes’ functional abundance was adjusted to the level of the control group, whereas the self-healing groups (S7 and S14) were adjusted to some extent through self-healing; however, it was not adjusted to the level of the control group. Among them, the relative abundance of genes related to metabolism is the highest in all samples, and its specific gene functions need to be further explored.

At level 2 (Figure 7B), further analysis of specific related genes functions (Table 4) shows that the relative abundance of genes functions such as global and overview maps, carbohydrate metabolism, amino acid metabolism, membrane transport, and energy metabolism in the model group is significantly higher than that in the control group, whereas the relative abundance of gene functions such as cell motility and immune system is significantly lower. On the 7th day, compared with the model group, the relative abundance of genes related to metabolism of intestinal flora in experimental group and self-healing group was regulated to some extent, but the regulation degree was poor compared with the control group. On the 14th day, the relative abundance of genes related to intestinal flora metabolism of mice in self-healing group (S14) was close to that of the control group, but the experimental group was adjusted to the level of the control group. Therefore, *Lactiplantibacillus plantarum* 2-33 can cause the dynamic change of the relative abundance of genes related to the metabolism of intestinal flora in AAD mice and then affect the regulation of bacterial diversity of intestinal flora.

**TABLE 4:** Predicting metabolic function genes based on PICRUSt2 analysis.

Pathway level 1	Pathway level 2
Metabolism	Global and overview maps
	Carbohydrate metabolism
	Amino acid metabolism
	Energy metabolism
	Metabolism of cofactors and vitamins
	Nucleotide metabolism
	Lipid metabolism
	Glycan biosynthesis and metabolism
	Biosynthesis of other secondary metabolites
	Metabolism of other amino acids
	Xenobiotics biodegradation and metabolism
	Metabolism of terpenoids and polyketides
Genetic information processing	Translation
	Replication and repair
	Folding, sorting and degradation
Environmental information processing	Signal transduction
Cellular processes	Cellular community - prokaryotes
	Cell growth and death
	Cell motility
	Transport and catabolism
Organismal systems	Endocrine system
	Aging
	Immune system
	Environmental adaptation
Human diseases	Drug resistance: antimicrobial
	Infectious disease: bacterial
	Cancer: overview
	Drug resistance: antineoplastic
	Endocrine and metabolic disease
	Infectious disease: viral

## Discussion

Intestinal flora is an interactive complex ecosystem, and its species and types have a great impact on the health of the host (28). Moreover, it has a variety of basic functions, including carbohydrate metabolism, interaction with the immune system, and prevention of pathogen invasion (29). However, the abuse of antibiotics will lead to the increase of drug resistance of some bacteria, which in turn will upset the balance of intestinal flora and lead to a series of adverse reactions such as diarrhea (30). Antibiotics will cause lasting and profound changes in the composition and function of intestinal flora, and it is difficult to return to the previous state (31). Because gentamicin sulfate is a broad-spectrum aminoglycoside drug, it has strong antibacterial activity against Gram-negative bacteria. Cefradine is a broad-spectrum cephalosporin of β-lactam, which has bactericidal effect on both Gram-positive and Gram-negative bacteria (20). Repeated use of these two antibiotics will upset the balance of intestinal flora and lead to the side effects similar to antibiotic-associated diarrhea (AAD). Therefore, to study the regulating effect of strain 2-33 on the intestinal flora imbalance of AAD mice, we established AAD mice model by gavage of the mixed antibiotic solution of gentamicin sulfate and cefradine, which caused the intestinal flora imbalance. In this study, mice were given mixed antibiotic solution by gastric perfusion for 5 days, which induced the obvious changes in diarrhea score and physiological characteristics of mice. That is to say, the diarrhea score of mice reached the highest value, and the mice showed sluggish movement, soft or watery feces, increased defecation times, sharp weight reduction, decreased food intake, and increased water intake, so it was determined that the AAD mice model was successfully established. Moreover, by comparing and analyzing the changes of body weight, food intake, and water intake of mice in experimental group and self-healing group, it is determined that strain 2-33 has a positive regulatory effect in curing diarrhea in AAD mice, and the gastric perfusion dosage has a significant effect on the speed of curing diarrhea.

The inflammatory response of the body is usually caused by the imbalance of cytokines (32), among which interleukin (IL) and tumor necrosis factor (TNF) play an important role in the immune system as communication factors between immune cells, which can reflect the inflammatory status of the host (33). Studies have shown that AAD is always accompanied by systemic inflammation, showing an increase in proinflammatory cytokines and a decrease in anti-inflammatory cytokines (34). In the model group of this study, the levels of anti-inflammatory cytokines such as IL-4 and IL-10 decreased significantly, whereas the levels of proinflammatory cytokines such as TNF-α and IFN-γ increased significantly (*p* < 0.05), which finally led to the increase of inflammatory cytokines in AAD mice, further demonstrating the successful establishment of AAD mice model. The level of anti-inflammatory cytokines and proinflammatory cytokines in the experimental group was adjusted to the level of the control group by gastric perfusion of strain 2-33 to the 14th day, which indicated that strain 2-33 had an important regulatory effect on the level of inflammatory cytokines in AAD mice serum. It is similar to probiotics *Bacillus* sp. DU-106 (35), *Lactobacillus reuteri* I5007 (36), and *Lactobacillus sakei* K040706 (37) in regulating cytokine levels in diarrhea mice. This provides a basis for the clinical application of the strain 2-33 after days.

The Ace index and Chao index in the alpha diversity of intestinal flora of AAD mice were significantly increased by gastric perfusion of strain 2-33 (*p* < 0.05). The results showed that the total number and relative abundance of intestinal flora increased, and the stability of intestinal flora increased (38). At the same time, PCoA and NMDS analysis of beta diversity showed that on the 7th day, the intestinal flora of experimental group and self-healing group tended to be in the same area, while on the 14th day, the intestinal flora of experimental group and control group gathered in the same area with a smaller area. The analysis showed that the intestinal flora of AAD mice in experimental group changed dynamically and finally adjusted to a normal state, and the days and doses of gastric perfusion had significant effects on the intestinal flora regulation of AAD mice. Based on the above analysis, we prove that strain 2-33 has a positive regulatory effect on the relative abundance and diversity of intestinal flora in AAD mice. Because a stable intestinal flora is a prerequisite for the host to resist the invasion of pathogenic bacteria and perform various biological functions, the imbalance of flora may pose a great threat to the health of the host and affect its physiological functions (39, 40). More and more evidence shows that probiotics can inhibit the proliferation of harmful bacteria in intestinal tract, promote the proliferation of beneficial bacteria, effectively regulate and balance intestinal flora, and help prevent and treat AAD (41). Therefore, strain 2-33 can positively regulate the intestinal flora balance of AAD mice.

The change of intestinal flora is related to the development of intestinal diseases, the ratio of Firmicutes and Bacteroidota is usually used to evaluate various intestinal diseases of patients, such as diarrhea, irritable bowel syndrome, and metabolic diseases and significantly affects the main components of intestinal flora, and this change is mainly caused by the destruction of intestinal microenvironment (35, 42). In this study, it was found that the relative abundance of Firmicutes increased, the relative abundance of Bacteroidota decreased, the ratio of Firmicutes and Bacteroidota increased significantly (*p* < 0.05), and the relative abundance of phylum also increased in the experimental group after the 14th day gastric perfusion of strain 2-33 compared with the self-healing group. Therefore, strain 2-33 has a positive effect on the regulation of intestinal flora diversity of AAD mice, and it reflects that the intestinal flora of mice is in a dynamic change of interaction and mutual influence, and the number of days and dosage of gastric perfusion have significant effects on the regulation of intestinal flora diversity of AAD mice. The results of this study are similar to those of Li (43) on the effect of *Astragalus membranaceus* polysaccharides on intestinal flora diversity of AAD diarrhea mice. Firmicutes are usually the representative of the intestinal flora of healthy individuals and may decrease with the occurrence of diseases (44). They contain a large number of Gram-positive bacteria, such as *Lactococcus, Lactobacillus, Listeri*, and so on, most of which are considered beneficial bacteria, helping to maintain the intestinal flora balance and prevent pathogen invasion (45); However, Bacteroidota is composed of common intestinal disease-related microorganisms, which have a relatively high risk of diarrhea and other diseases and are negatively correlated with inflammatory cytokines (46). It has been reported that Firmicutes and Bacteroidota play an important role in the digestion of carbohydrates and protein, which is conducive to the maturation of intestinal immune system, and the high relative abundance of Firmicutes and Bacteroidota in intestinal flora may be closely related to the energy and nutritional requirements of the host (47). Because strain 2-33 can increase the proportion of Firmicutes and Bacteroidota in the intestinal flora of AAD mice, thus regulating the diversity of intestinal flora of AAD mice, and then curing diarrhea of mice, the intestinal flora of AAD mice on the 7th day and 14th day is significantly different at the genus level, and the dynamic changes in intestinal flora may play a key role in the intestinal ecosystem and function. Compared with the self-healing mice, strain 2-33 increased the relative abundance of *Lactobacillus* in the experimental group and decreased the relative abundance of *Enterococcus*. *Lactobacillus* has long been regarded as a beneficial intestinal bacterium, which plays an important role in maintaining the microecological balance of intestinal flora, reducing the colonization of pathogenic organisms, and improving growth performance (22). It can promote the development of T helper cells, induce the production of cytokines, and enhance cellular immune function. The decrease of relative abundance of *Lactobacillus* means that colonization resistance and intestinal mucosal barrier are weakened (48). In addition, *Lactobacillus* can enhance intestinal antioxidant capacity and digestive enzyme activity (49). However, the change of *Enterococcus* is particularly prominent, and the relative abundance of *Enterococcus* in the model group is significantly increased (*p* < 0.05), which is related to the pathological changes in intestinal mucosa in AAD mice (48). Moreover, *Enterococcus* is an important pathogen, with *E. faecalis* and *E. faecium* as the most representative strains, which can cause a variety of infections. Many *Enterococcus* have plasmid-encoded drug-resistant genes, and these genes lead to a decrease in intrinsic sensitivity to several antibacterial drugs such as gentamicin and cefradine (50, 51); Moreover, *Enterococcus* has a variety of virulence factors, which are involved in mediating adhesion, colonization, and invasion of host tissues, regulating host immunity, and extracellular production of enzymes and toxins (52). Therefore, in this study, *Enterococcus* showed intrinsic resistance to gentamicin and cefradine, which were not inhibited or killed, but proliferated in large numbers. Therefore, the relative abundance of *Enterococcus* in the model group accounted for about 99%; *Bacteroides* are important clinical pathogens, which are resistant to many antibiotics and affect the host immune system (53). Therefore, a large number of *Bacteroides* appeared in the experimental group and the self-healing group on the 7th day, but by 14th day, the relative abundance of *Bacteroides* decreased rapidly and reached the control level. To sum up, strain 2-33 can adjust the structure and relative abundance of intestinal flora of AAD mice, increase the diversity of flora, inhibit or kill typical diarrhea-related bacteria, and increase the relative abundance of intestinal beneficial bacteria and normal bacteria. Probiotics can inhibit intestinal pathogens by producing antibacterial compounds, compete for rejection by consuming limited nutritional resources or adhering to epithelial cells, or stimulate intrinsic microbial activity (2). This is consistent with the research results of Zhang (54) that *Lactiplantibacillus plantarum* HOM3204 can significantly increase the relative abundance of *Lactobacillus* in AAD mice and decrease the relative abundance of *Enterococcus*. At the same time, we also found that the efficacy of strain 2-33 on AAD mice was time- and dose-dependent, which was similar to the results of Ling’s (55) study on the efficacy of *Clostridium butyricum* and *Bifidobacterium infanti* on AAD mice. Therefore, it is fully proved that strain 2-33 has a positive effect on the regulation of intestinal flora diversity of AAD mice, and the number of days and dosage of gastric perfusion have effects.

Because the intestinal flora is considered to be an organ that helps to regulate the metabolism of the host, the change of intestinal flora leads to the change of metabolic pathway, and the most obvious one is that the amino acid metabolism will obviously increase in AAD mice (34). Moreover, amino acid can enhance cell metabolism, increase the synthesis of protein by regulating protein translation, and increase the content of mitochondria in skeletal muscle and adipocytes, which is essential to maintain normal physiological internal environment balance (56). Carbohydrate metabolism can regulate cellular processes and provide material basis for other metabolic pathways (57). In this study, strain 2-33 can regulate global and overview maps, carbohydrate metabolism, and amino acid metabolism in AAD mice of experimental group to the level of control group. However, the basic metabolic function of microorganisms is predicted based on 16S rRNA genes, so it is necessary to further confirm the bacterial metabolism of each sample at the genome level through metagenome sequencing (58).

In summary, strain 2-33 has a positive regulatory effect on restoring physiological indexes and inflammatory cytokine levels of AAD mice, regulating intestinal flora diversity and metabolism-related functional genes. Therefore, the results provide a basis for the application of strain 2-33 in antibiotic-associated diarrhea.

## Conclusion

Diarrhea is a common multifactorial gastrointestinal disease, characterized by thin stool and increased water content. It is a potentially fatal disease all over the world. Most diarrhea is now treated with antibiotics, but the abuse of antibiotics seriously damages the diversity, consistency, and functionality of intestinal flora, so it is of great significance to prevent diarrhea and treat diarrhea with non-drugs. In this study, the mixed antibiotic solution of gentamicin sulfate and cefradine significantly changed the relative abundance and diversity of intestinal flora in mice, which led to the imbalance of intestinal flora and diarrhea and finally successfully established the AAD mice model. Gastric perfusion of strain 2-33 to AAD mice can regulate various physiological indexes, significantly increase the levels of anti-inflammatory factors IL-4 and IL-10, and significantly decrease the levels of proinflammatory factors TNF-α and IFN-γ (*p* < 0.05). At the same time, strain 2-33 has a positive regulating effect on the intestinal flora balance of AAD mice, especially at the genus level, and it can increase the relative abundance of *Lactobacillus* and decrease the relative abundance of *Enterococcus* and *Bacteroides*. In addition, carbohydrate metabolism, amino acid metabolism, energy metabolism, and so on can be adjusted to normal levels, thus promoting the regulation of intestinal flora structure. However, the regulation effect of strain 2-33 on intestinal flora of AAD mice is time- and dose-dependent, that is, short-term gastric perfusion and low dose have no significant effect on the regulation of intestinal flora diversity (*p* > 0.05), but with the extension of gastric perfusion days and the increase of gastric perfusion dose, it has a significant effect on the regulation of intestinal flora diversity (*p* < 0.05). In summary, *Lactiplantibacillus plantarum* 2-33 can regulate the composition and diversity of intestinal flora in mice with antibiotic-associated diarrhea and promote the reconstruction of intestinal flora environment, thus alleviating and curing diarrhea symptoms.

## Data Availability Statement

The datasets presented in this study can be found in online repositories. The names of the repository/repositories and accession number(s) can be found in the article/supplementary material.

## Ethics Statement

The animal study was reviewed and approved by the Experimental Animal Welfare and Ethics Committee of Inner Mongolia Agricultural University. Written informed consent was obtained from the owners for the participation of their animals in this study.

## Author Contributions

WB, YH, and JY designed the experiments. XY and ML conducted most of the experiments. WB, YH, and CL wrote and edited the manuscript. EZ and NT reviewed the manuscript. All authors read and approved the manuscript.

## Conflict of Interest

The authors declare that the research was conducted in the absence of any commercial or financial relationships that could be construed as a potential conflict of interest.

## Publisher’s Note

All claims expressed in this article are solely those of the authors and do not necessarily represent those of their affiliated organizations, or those of the publisher, the editors and the reviewers. Any product that may be evaluated in this article, or claim that may be made by its manufacturer, is not guaranteed or endorsed by the publisher.
